# Two different domain architectures generate structural and functional diversity among *bZIP* genes in the Solanaceae family

**DOI:** 10.3389/fpls.2022.967546

**Published:** 2022-08-19

**Authors:** Jin-Wook Choi, Ha-Eun Kim, Seungill Kim

**Affiliations:** Department of Environmental Horticulture, University of Seoul, Seoul, South Korea

**Keywords:** *bZIP*, transcription factors, Solanaceae, abiotic stress, integrated domain, re-annotation

## Abstract

The *bZIP* gene family is one of the largest transcription factor families and has important roles in plant growth, development, and stress responses. However, *bZIP* genes in the Solanaceae family have not been extensively investigated. Here, we conducted genome-wide re-annotation in nine Solanaceae species and *Arabidopsis thaliana*. We annotated 935 *bZIP* genes, including 107 (11%) that were newly identified. Structural analyses of *bZIP* genes in the Solanaceae family revealed that the bZIP domain displayed two types of architectures depending on the presence of an additional domain, suggesting that these architectures generate diversified structures and functions. Motif analyses indicated that the two types of *bZIP* genes had distinct sequences adjacent to the bZIP domain. Phylogenetic analyses suggested that the two types of *bZIP* genes distinctly evolved and ultimately adapted in different lineages. Transcriptome analyses in pepper (*Capsicum annuum*) and tomato (*Solanum lycopersicum*) revealed putative functional diversity between the two types of *bZIP* genes in response to various abiotic stresses. This study extensively updated *bZIP* gene family annotations and provided novel evolutionary and functional evidence for the role of *bZIP* genes in Solanaceae plants. Our findings provide evolutionary and functional characteristics of *bZIP* genes for a better understanding of their roles in Solanaceae plants.

## Introduction

Plants have evolved sophisticated mechanisms regulated through multiple gene families to maintain optimal growth under changing environments in response to a variety of stresses (Scharf et al., 2012). Transcription factors (TFs) belong to one of the regulator gene families that has a role in regulating transcription by attaching to the *cis*-element of a target gene promoter region (Mitchell and Tjian, 1989; Talanian et al., 1990). The bZIP TF gene family contains the bZIP DNA-binding domain, which contains 40–80 amino acids that include a basic region, a leucine zipper region, and an interconnecting hinge region (Landschulz et al., 1988; Perez-Rodriguez et al., 2010). The basic region is conserved, consists of 16–18 amino acids, and contains an invariant N-X_7_-R/K motif for binding to specific DNA sequences with an ACGT core, such as A-box (TACGTA), C-box (GACGTC), and G-box (CACGTG; Kouzarides and Ziff, 1989; Izawa et al., 1993; Deppmann et al., 2004). The leucine zipper region is composed of heptad repeats of leucine or other hydrophobic amino acids, which mediates the dimerization of bZIP protein (Landschulz et al., 1988; Ehlert et al., 2006; Llorca et al., 2015). The *bZIP* gene family has been identified and studied in plants including *Arabidopsis thaliana* (Jakoby et al., 2002), *Oryza sativa* (Nijhawan et al., 2008), *Zea mays* (Wei et al., 2012), *Vitis vinifera* (Liu et al., 2014b), and six species of legume (Wang et al., 2015). Functional studies of the *bZIP* gene family indicate that it has a key role in plant growth, development, and response to biotic/abiotic stresses (Uno et al., 2000; Baena-Gonzalez et al., 2007).

More than 3,000 plant species belong to the Solanaceae family, including many economically important crops such as *Capsicum annuum* and *Solanum lycopersicum* (Rigano et al., 2013). *Capsicum annuum* is a major ingredient in spicy cuisine and provides essential dietary vitamins and minerals (Kim et al., 2019b). *Solanum lycopersicum* contains many nutrients that promote human health, such as carotenoids and anthocyanins, and is widely used in genomic research because of its small genome size and short generation time (Rigano et al., 2013). Whole-genome sequencing of Solanaceae crops has been completed, thereby providing opportunities to explore the *bZIP* gene family in individual species of the Solanaceae family, such as *S. lycopersicum*, *Solanum tuberosum*, and *C. annuum* (Li et al., 2015; Gai et al., 2020; Herath and Verchot, 2020; Wang et al., 2021). Despite the availability of Solanaceae genomic resources, comparative genomics and transcriptomics analyses of *bZIP* genes in the Solanaceae family have not yet been performed.

In this study, we re-annotated and comparative analyzed *bZIP* genes in nine Solanaceae species along with *A. thaliana*. We identified 935 *bZIP* genes, including 107 (11%) updated genes, which were used in our further analysis. The overall structural features of *bZIP* genes identified two bZIP domain architectures in the Solanaceae family. Extensive motif analyses showed that the bZIP domains of the two types of *bZIP* genes contained distinct sequence compositions. Phylogenetic analysis indicated that *bZIP* genes were clustered into 14 subgroups, including 13 subgroups previously known in *A. thaliana* and 1 newly constructed subgroup with distinct domain architecture. Expression analyses incorporating gene ontology (GO) enrichment data suggested that *bZIP* genes have diverse functions in pepper (*C. annuum*) and tomato (*S. lycopersicum*) under abiotic stress conditions. Our study provided comprehensive information on the structure, expression, and functions of *bZIP* genes in Solanaceae. These results would be useful for future agricultural studies in Solanaceae crops.

## Materials and methods

### Re-annotation of *bZIP* gene family in 10 plant genomes

To re-annotate the *bZIP* gene family in 10 plant species, we downloaded the genomic and transcriptomic data for the following plants: *A. thaliana* (Lamesch et al., 2012), *Nicotiana benthamiana* (Bombarely et al., 2012), *Petunia inflata* (Bombarely et al., 2016), *C. annuum* (Kim et al., 2014), *Capsicum chinense* (Kim et al., 2017), *Capsicum baccatum* (Kim et al., 2017), *S. tuberosum* (Pham et al., 2020), *S. lycopersicum* (Fernandez-Pozo et al., 2015), *Solanum pennellii* (Bolger et al., 2014), and *Solanum pimpinellifolium* (Wang et al., 2020; Supplementary Table 1). We used TGFam-Finder v1.20 for re-annotation of *bZIP* genes considering the parameters described previously (Kim et al., 2020). The TSV files including functional domain information were generated by InterProScan 5 (-f tsv-appl Pfam; Jones et al., 2014) and used as “TSV_FOR_DOMAIN_IDENTIFICATION.” The “TARGET_DOMAIN_ID” was set as “PF00170 (bZIP)” according to the Pfam database.^1^

We newly assigned gene names to re-annotated *bZIP* genes instead of using locus tag names from the published annotations. We matched gene names to previously annotated names if they had been assigned in previous research, as for *bZIP* genes in *A. thaliana*, *C. annuum*, *S. lycopersicum*, and *S. tuberosum* (Jakoby et al., 2002; Li et al., 2015; Gai et al., 2020; Herath and Verchot, 2020; Supplementary Table 2). New names also were assigned to the updated *bZIP* genes from the other species.

### Domain structures of *bZIP* genes

The domain architectures of the updated *bZIP* genes were analyzed using TSV files generated by InterProScan 5 (-f tsv-appl Pfam; Jones et al., 2014) according to the Pfam database.^1^ We defined integrated domains (IDs) if *bZIP* genes had other domain (s) in addition to the bZIP domain (PF00170). To acquire more precise information, we excluded domains with high e-value (>1e − 4) or those that overlapped the bZIP domain.

### Surveying amino acid sequence composition of the bZIP domain

We extracted the bZIP domain sequences of the 10 plant genomes to determine the amino acid sequence compositions of the bZIP domains. We utilized MAFFT v7.470 (Katoh and Standley, 2013) to align the bZIP domain sequences, and then trimmed the alignment with TrimalAl v1.4 (-gt 0.5; Capella-Gutierrez et al., 2009). WebLogo v2.8.2^2^ (Crooks et al., 2004) was used for the visualization of amino acid sequence composition. We divided the bZIP domain into five compartments based on signature residues. The conservation score of each compartment was determined as the average of the scores of residues within each compartment as calculated by CLC Sequence Viewer software v8.0.

### Gene ontology analysis

We performed a GO analysis to analyze the putative function (s) of *bZIP* genes using OmicsBox v1.4.^3^ BLASTP was used to align bZIP protein sequences to the NCBI non-redundant protein database (nr v5) with e-value cut off 10^−3^. The results of InterProScan (Jones et al., 2014) results were integrated with the BLAST results. Next, we performed Blast2GO Mapping and Blast2GO Annotation with default parameters. The results of GO analysis were grouped into three categories (biological process, molecular function, and cellular component). We displayed the top five GO terms in the direct GO count of each category in the analysis.

### Motif analysis of *bZIP* genes

To search conserved motifs of all protein sequences of *bZIP* genes, we used the MEME v5.1.1 (Bailey et al., 2006) program with the following parameters: -mod zoops, -nmotifs 50, -minw 10, -maxw 50, -objfun se, -markov_order 0. We used MAST v5.1.1 to match protein sequences to set of motifs (Bailey and Gribskov, 1998). We decided the position of conserved motifs manually using sequence alignments and motif compositions. To clarify the motif position of the top five gene structures, we excluded motifs that were repetitively placed at various motif sites.

### Statistical enrichment test

We performed an enrichment test of motifs using Fisher’s test and Chi-square test functions from the Statistics::R module in R to check whether specific motifs were enriched in genes that contain a bZIP domain only or IDs. *p*-values were calculated by Monte Carlo test (B = 10,000). *p*-values < 0.0001 were regarded as highly important for the assured enrichment test.

### Phylogenetic analysis of *bZIP* genes

Multiple sequence alignments of 935 re-annotated bZIP protein sequences were performed using MAFFT v7.470 (Katoh and Standley, 2013). TrimAL v1.4 was used to eliminate ambiguous alignments using the gt 0.5 trimming option (Capella-Gutierrez et al., 2009). The maximum-likelihood phylogenetic tree was generated using IQ-TREE v2.0.6 with JTT + R6 amino acid substitution model and 1,000 ultrafast bootstrap replicates (Minh et al., 2020). To visualize the mid-point rooted tree, we used Interactive Tree of Life (iToL) v6.^4^ The tree of *bZIP* genes was clustered into 14 subgroups (13 previously assigned subgroups and one unassigned subgroup) based on domain and motif structures. We named the unassigned clade as the St subgroup because most of the genes in the St subgroup contained the StAR-related lipid-transfer (START) domain.

### Transcriptome and GO enrichment analysis

We investigated the expression profiles of the *bZIP* gene in *C. annuum* and *S. lycopersicum* using *C. annuum* (Kang et al., 2020) and *S. lycopersicum* RNA-seq data in leaf under various abiotic stresses (SRR7652567, SRR7652566, SRR7652565, SRR7652564, SRR7652571, SRR7652570, SRR7652569, SRR7652568, SRR7652563, SRR15410554, SRR15410555, SRR15410556, SRR15410551, SRR15410552, SRR15410553, SRR15607561, SRR15607560, SRR15607558, SRR15607557, SRR15607556, and SRR15607555). RNA-seq data in *C. annuum* were generated under cold, heat, osmotic, and salt stress treatments at different time points (3, 6, 12, 24, and 72 h). RNA-seq data in *S. lycopersicum* were generated under cold, heat, drought, and salt stress treatments without reference to specific time points. All experiments were performed with three biological repeats. We trimmed the raw FASTQ files using CLC Assembly Cell (CLC Bio, Aarhus, Denmark) to eliminate low-quality data. Filtered data were mapped to the *C. annuum* and *S. lycopersicum* to reference genomes using HISAT2 (-dta-x; Kim et al., 2019a). We performed StringTie (-e-B-G; Pertea et al., 2015) to calculate fragment per kilobase of transcript per million mapped reads (FPKM) values of whole genes with the newly annotated *bZIP* genes in *C. annuum* and *S. lycopersicum*. FPKM values were converted to read counts using python scripts (prepDE.py). Differentially expressed genes (DEGs) were identified using DESeq2 in R software with the following criteria: log_2_FoldChange > 1 or < −1, and adjusted *value of p* <0.05 (Love et al., 2014).

We performed expressional clustering analysis of all *C. annuum* DEGs including bZIP DEGs. We grouped all *C. annuum* DEGs by expression patterns under all abiotic stresses at different time points using the Mfuzz program in R software (Kumar and Futschik, 2007). Four clusters were identified using the k-means algorithm. We also grouped all *S. lycopersicum* DEGs into upregulated or downregulated groups under each stress regime. Then, we conducted GO annotation in each cluster/group using OmicsBox v1.4.^5^ We examined the significance of the GO enrichment analysis using Fisher’s exact test (false discovery rate adjusted *value of p* ≤ 0.01) in each cluster/group.

## Results and discussion

### Comprehensive characteristics of updated *bZIP* genes in Solanaceae

**Figure 1:**
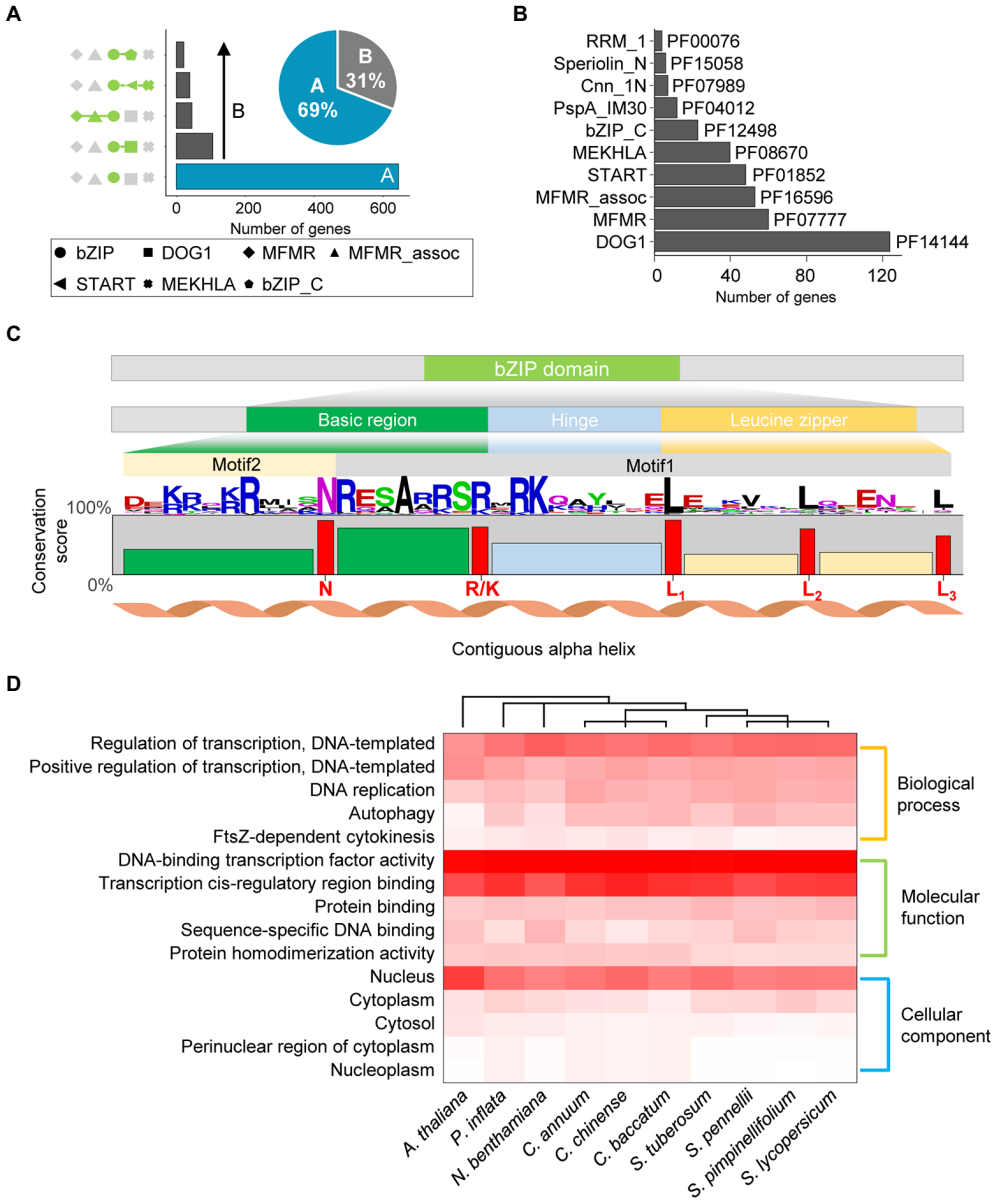
Characteristics of *bZIP* genes in Solanaceae. **(A)** The number of genes with the top five domain architecture repertoires is illustrated in the bar chart. Different domain architectures are shown on the left of the chart; domains are defined by different shaped symbols as defined below the chart. The percentages of the two types of *bZIP* genes are presented in the pie chart. **(B)** The bar chart shows the number of genes with integrated domains (top 10). Pfam IDs of the integrated domains are labeled next to the chart. **(C)** The amino acid sequence of the bZIP domain in 10 species. The height of the amino acid residue indicates the relative frequency of each amino acid at the specific position. The conservation scores of each compartment and residues are displayed as bar plots. The bar colors represent three regions of the bZIP domain: green, basic region; light blue, hinge region; and yellow, leucine zipper region. Red letters below the bar plots represent significantly conserved residues in *bZIP* genes. **(D)** Distribution of gene ontology (GO) terms of *bZIP* genes. The three GO categories are displayed on the right side of the heat map. The top five GO descriptions in each category are shown in the heat map. The colored scale at the bottom right side of the heat map represents the proportion of *bZIP* genes in each species.

We re-annotated the *bZIP* genes in nine Solanaceae species and *A. thaliana* to construct improved *bZIP* gene models. We identified 935 *bZIP* genes in 10 plant genomes (Table 1), and 107 (11%) of these genes were newly annotated. The number of *bZIP* genes per genome ranged from 69 (*C. annuum*) to 193 (*N. benthamiana*), and the number of newly annotated *bZIP* genes per genome ranged from 1 (*S. tuberosum*) to 32 (*N. benthamiana*). We examined domain architectures in the updated genes to explore the genome structure of *bZIP* genes in the 10 plant genomes. Our analysis revealed that *bZIP* genes primarily displayed two types of domain architectures: 645 *bZIP* genes (69%) contained only the bZIP domain (bZIP_only), and 290 *bZIP* genes (31%) contained additional integrated domains (bZIP_IDs; Figure 1A). The proportion of *bZIP* genes displaying these two architectures was similar in 8 of the species, whereas *N. benthamiana* and *A. thaliana* had slightly higher proportions of the bZIP_only architecture (74%; Supplementary Figure 1). Among *bZIP* genes displaying the bZIP_IDs architecture, 124 *bZIP* genes contained the DOG1 (PF14144) domain (42% of bZIP_IDs), which is involved in controlling seed dormancy (Figure 1B; Bentsink et al., 2006). Other less abundant bZIP_IDs domains included MFMR (PF07777), MFMR_assoc (PF16596), START (PF01852), MEKHLA (PF08670), and bZIP_C (PF12498; Figure 1B). These results provide the domain architecture repertoire of *bZIP* genes in the Solanaceae family and *A. thaliana* based on our updated annotations.

**Table 1:** Numbers of re-annotated *bZIP* genes in nine Solanaceae species and *Arabidopsis thaliana*.

Species	Previously annotated genes	Newly annotated genes	Total
*Arabidopsis thaliana*	78	3	81
*Petunia inflata*	76	21	97
*Nicotiana benthamiana*	161	32	193
*Capsicum annuum*	64	5	69
*Capsicum chinense*	68	5	73
*Capsicum baccatum*	65	9	74
*Solanum tuberosum*	86	1	87
*Solanum pennellii*	83	8	91
*Solanum pimpinellifolium*	74	11	85
*Solanum lycopersicum*	73	12	85
Total	828	107	935

We investigated the amino acid sequence composition of the bZIP domain in 10 plant species. The bZIP domain contains three motif regions (basic, hinge, and leucine zipper regions) that display N-X_7_-R/K, X_9_, and L-X_6_-L amino acid sequences, respectively (Droge-Laser et al., 2018). Our analysis shows that the bZIP domain in Solanaceae with *A. thaliana* was also clearly separated into three regions with those known signature residues of the bZIP domain covered by two conserved motifs (motif #2 and #1 in order; Figure 1C). This result supports the high accuracy of our updated annotation based on the known signature motifs and sequences in the bZIP domain. Comparative analysis of bZIP domains by species indicated that they were highly conserved (Supplementary Figure 2A), although bZIP domains significantly differed according to the major domain architectures (Supplementary Figure 2B). This result suggests that the distinct sequence compositions of bZIP domains with different domain architectures originated from the independent evolution of each bZIP domain architecture. We divided the bZIP domain into five compartments based on their highly conserved residues (N, R/K, L_1_, L_2_, and L_3_) and calculated the amino acid conservation score for those residues in each compartment (Figure 1C). The conservation scores of the highly conserved residues (N, R/K, L_1_, L_2_, and L_3_) and the second compartment (located between the N and R/K regions) were significantly higher (64%–90%) than those of the first, third, fourth, and fifth compartments (39%, 49%, 31%, and 34%, respectively). These results indicate that this compartment and residues were highly conserved in the Solanaceae family, which was consistent with previous reports (Li et al., 2015, 2021; Gai et al., 2020; Wang et al., 2021).

To characterize the putative function (s) of the updated *bZIP* genes, we performed GO analysis (Figure 1D). GO terms of 935 *bZIP* genes were classified into three categories: biological process, molecular function, and cellular component. The overall distributions of GO terms were similar for each species (Figure 1D). The most dominant GO terms in the three categories were “Regulation of transcription” (58%), “DNA-binding transcription factor activity” (95%), and “Nucleus” (56%), respectively. This suggests potential functions of updated *bZIP* genes as TFs in Solanaceae, as the *bZIP* gene family contained known TFs involved in plant development (Izawa et al., 1993). Taken together, our results showed that the updated annotation of *bZIP* genes enabled precise analyses of the domain structure, sequence composition, and putative function of *bZIP* genes in nine Solanaceae species and *A. thaliana*.

### Motif compositions of the bZIP_only and bZIP_IDs

**Figure 2:**
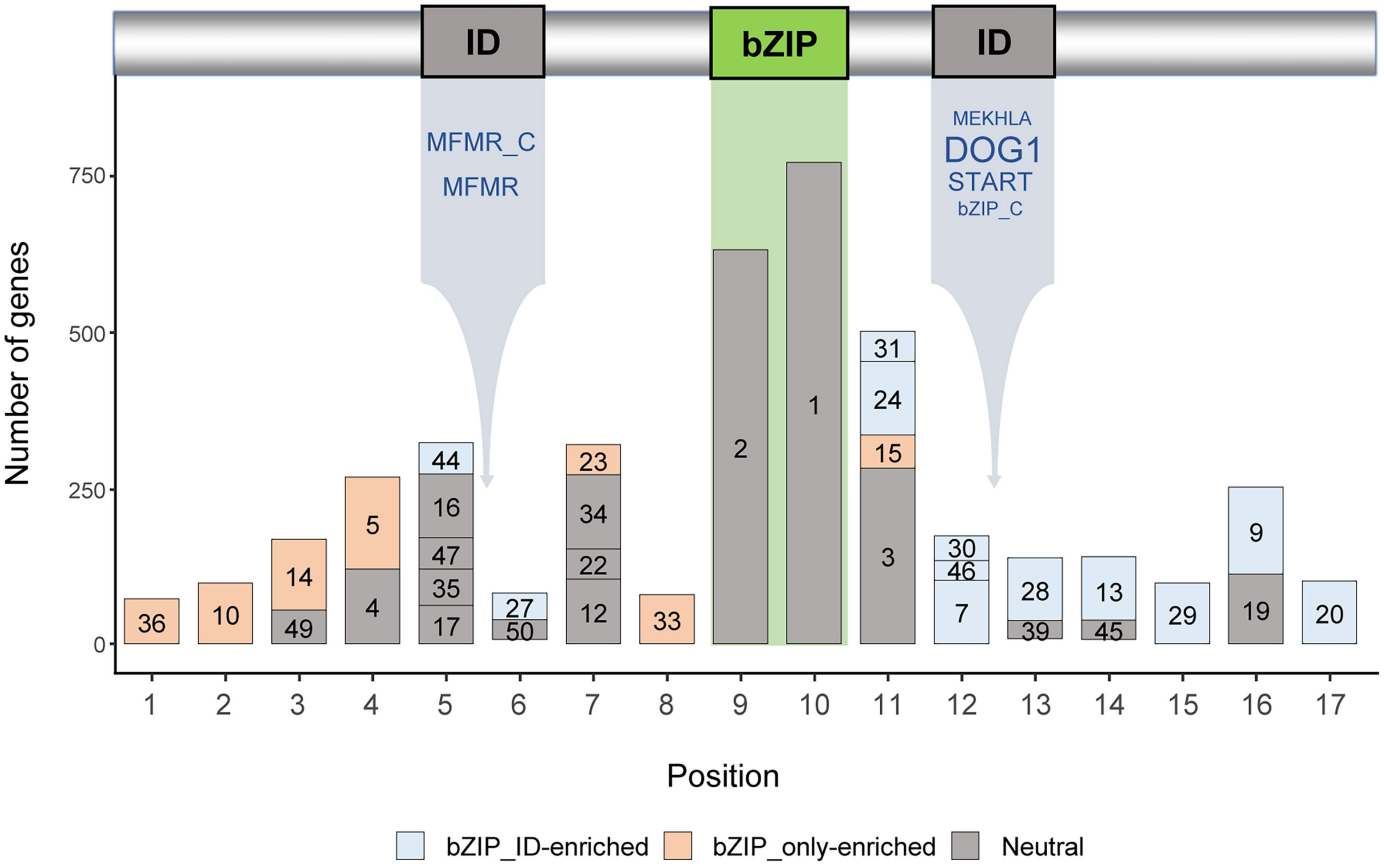
Motif compositions of the top five domain architectures of *bZIP* genes. The numbers in the bars indicate motif labels as described in Supplementary Table 2. The types of integrated domains are listed below the ID box. The font sizes of the names of integrated domains represent the number of domains. Bar colors represent bZIP_only-enriched, bZIP_IDs-enriched, or neutral (*p* < 0.0001).

Motifs that exist outside of the bZIP domain enhance the structural and functional diversity of *bZIP* genes (Nijhawan et al., 2008; Wei et al., 2012). We surveyed the updated *bZIP* genes and identified 50 conserved motifs, excluding 20 genes that did not contain any conserved motifs (Supplementary Table 3). We verified that 35 of the 50 motifs were located in 17 specific positions (Figure 2), whereas 15 of the 50 motifs were located in various positions and were excluded from further analysis. These results suggest that *bZIP* genes in Solanaceae contained a variety of sequence motifs encompassing the bZIP domain, thereby increasing the sequence diversity of *bZIP* genes as described in previous reports (Nijhawan et al., 2008; Wei et al., 2012; Liu et al., 2014b). We found that other domains in bZIP_IDs (Figure 1A) were located between positions #5 and #6 (MFMR and MFMR_C) or positions #12 and #13 (DOG1, START, MEKHLA, and bZIP_C), suggesting that other motifs occupied conserved locations in bZIP_IDs (Figure 2).

We performed enrichment tests of the 35 motifs in conserved locations to identify motifs that were abundant in bZIP_only or bZIP_IDs (Figure 2). We classified motifs into three groups: motifs enriched in bZIP_only, motifs enriched in bZIP_IDs, and motifs that were not enriched in any type of domain architecture. Our analyses showed that the motif compositions and locations of abundant motifs significantly differed in bZIP_only and bZIP_IDs. These results suggest that bZIP_only and bZIP_IDs distinctly evolved after the emergence of bZIP_IDs through domain integration into bZIP_only. Most of the enriched motifs in bZIP_only and bZIP_IDs were located upstream and downstream of the bZIP domain, respectively (Figure 2). This may indicate that bZIP_only primarily obtained specific sequences upstream of the bZIP domain, whereas bZIP_IDs gained specific downstream motifs through domain integration.

### Distinct phylogenetic lineages of *bZIP* genes

**Figure 3:**
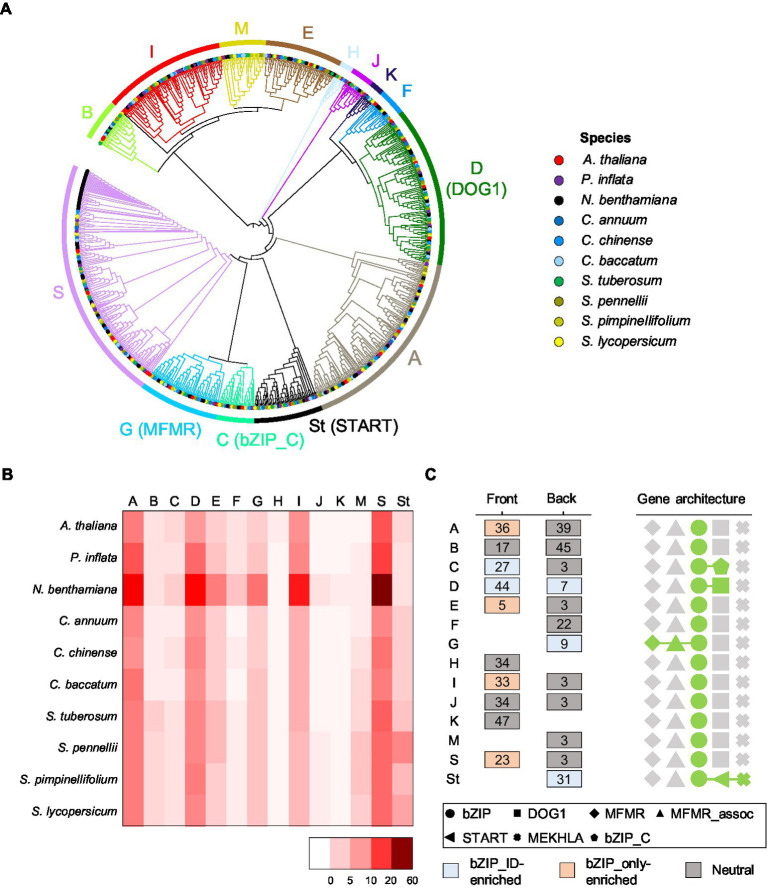
Phylogenetic relationships among *bZIP* genes and subgroup characteristics. **(A)** Phylogenetic tree of *bZIP* genes in the 10 plant species. Different species on the end of the branch are denoted by different colors. The colored branch and colored bars on the outer ring represent individual subgroups. Subgroup names are listed next to the outer rings representing integrated domains in the bracket. **(B)** The numbers of *bZIP* genes in subgroups by species are shown in a heatmap. Scale bars on the bottom right side indicate the number of genes. **(C)** The most abundant motifs adjacent to the bZIP domain are portrayed. Numbers in boxes indicate motif labels. The box colors represent the type of enriched motifs. The top five domain architecture repertoires are shown on the right side as different geometric patterns.

To explore the evolutionary relationships of Solanaceae *bZIP* genes (bZIP_only and bZIP_IDs), we constructed a phylogenetic tree using the updated *bZIP* genes in 10 species. We divided them into 14 subgroups, including 13 subgroups that were consistent with those described previously in *A. thaliana* (Droge-Laser et al., 2018) and 1 newly constructed subgroup (St) that was omitted in the previous phylogenetic tree (Li et al., 2015; Droge-Laser et al., 2018; Gai et al., 2020; Wang et al., 2021; Figure 3A). We found that bZIP_only and bZIP_IDs were distinctly clustered among the subgroups. The dominant bZIP_IDs were enriched in four subgroups: START domain in the St subgroup (81%), MFMR domain in subgroup G (92%), DOG1 domain in subgroup D (93%), and bZIP_C domain in subgroup C (74%). This suggests that those bZIP_IDs evolved through independent copy number expansion, and were finally adopted as individual lineages. We then examined the number of *bZIP* genes in each subgroup to verify the copy number variation of *bZIP* genes among different subgroups and among the same subgroups in different species (Figure 3B). The subgroup S containing 21% of the updated *bZIP* genes was the largest lineage, and I (11%), A (17%), and D (14%) were observed as dominant subgroups in order, suggesting copy number expansion of specific lineages. Including these subgroups, we found a similar proportion of *bZIP* genes in the 10 plant species belonging to the same subgroup (Figure 3B). This result suggests that the progenitor *bZIP* genes of Solanaceae emerged in a common ancestor of Solanaceae and Brassicaceae (Correa et al., 2008).

Previous studies reported distinct functions of *bZIP* genes depending on subgroup-specific motifs in *A. thaliana* (Choi et al., 2000; Lopez-Molina et al., 2001; Bensmihen et al., 2002). Subgroup A-specific motifs contain casein kinase II (CKII) phosphorylation sites of ABRE-binding factor (ABF) and ABA-responsive element-binding protein (AREB) genes. Both genes function as ABA-dependent TFs to control abiotic stress tolerance, which is a representative function of subgroup A. We investigated the motifs of *bZIP* genes in each subgroup, excluding motifs of the bZIP domain, to characterize sequences representing each subgroup (Figure 3C). The results showed that the ID-specific subgroups (subgroup C, D, G, and St) included bZIP_IDs-enriched motifs, whereas *bZIP* genes in other subgroups primarily containing bZIP_only had motifs enriched in bZIP_only or common motifs observed in bZIP_only and bZIP_IDs. Some motifs appeared in a specific group as subgroup-specific motifs; for example, motifs #36, #33, and #23 appeared specifically in subgroups A, I, and S, respectively (Figure 3C). These results and previous studies (Choi et al., 2000; Finkelstein and Lynch, 2000; Lopez-Molina et al., 2001; Bensmihen et al., 2002; Jakoby et al., 2002) suggest that distinct motifs enhanced the structural diversity and divergent functions of *bZIP* genes in each subgroup. Our analyses revealed the phylogenetic relationships and lineage-specific structural characteristics of *bZIP* genes in Solanaceae, which will facilitate future genetic and functional studies in agriculturally important crops.

### Expression and putative functional analyses of *bZIP* genes in pepper under abiotic stress

**Figure 4:**
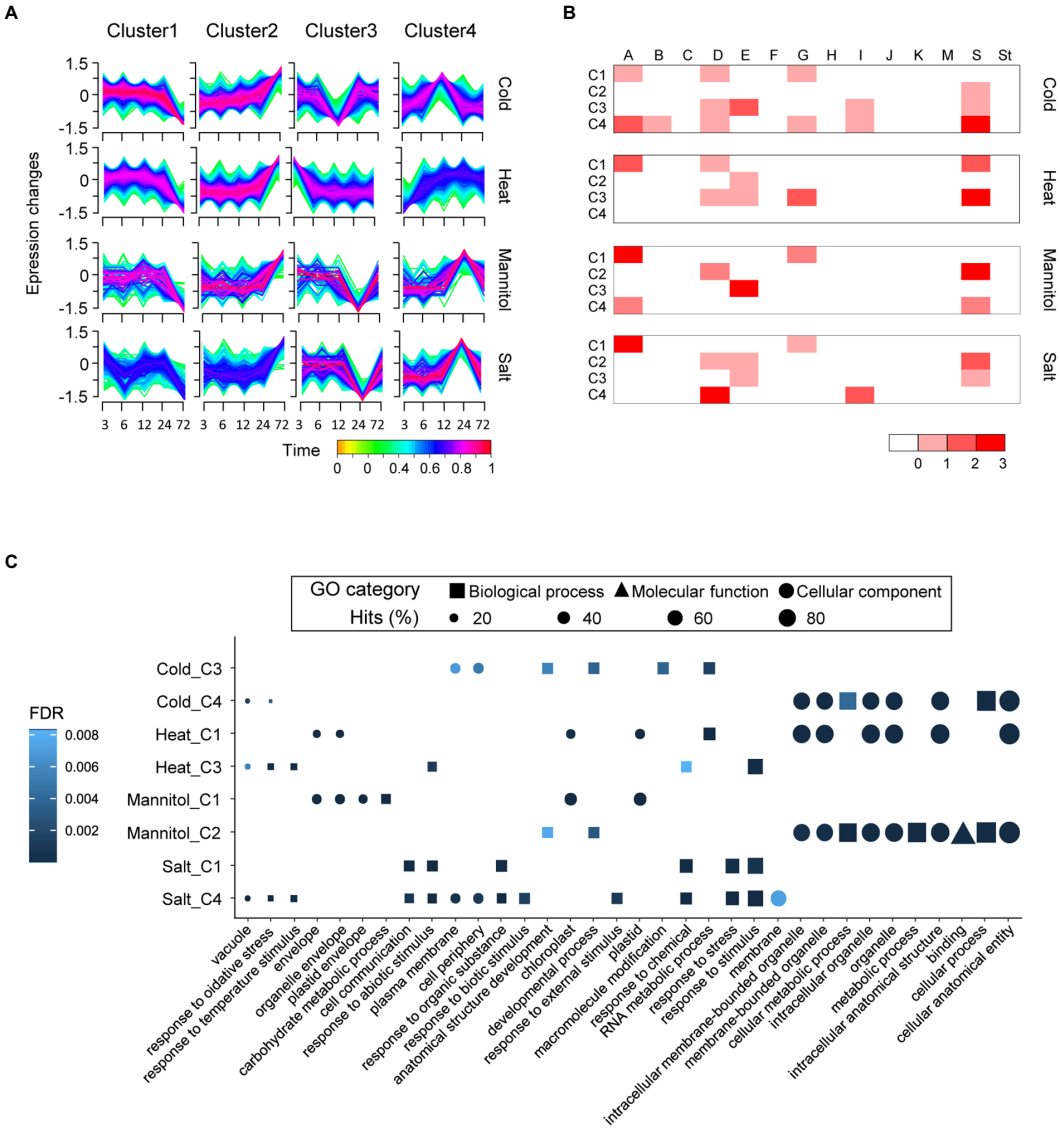
Transcriptome and gene ontology (GO) enrichment analyses of differentially expressed genes under four abiotic treatments in pepper (*Capsicum annuum*). **(A)** Expression patterns of whole pepper DEGs and bZIP DEGs during abiotic stress are displayed as four clusters for each abiotic stress. **(B)** Heatmap depicts the number of bZIP DEGs in each subgroup. The scale colors on the bottom right side of the heat map display the number of genes. **(C)** The top five GO terms enriched in each major cluster are displayed in dot plots. Shapes represent the GO categories; shape sizes represent the GO term frequencies.

The *bZIP* genes have crucial roles in response to abiotic stress (Hossain et al., 2010; Liu et al., 2014a). We conducted expression analyses of whole genes of pepper (*C. annuum*), including the newly identified *bZIP* genes, to examine the potential functions of *bZIP* genes under various abiotic stress conditions. The expression profiles were investigated under four stresses (cold, heat, mannitol, and salt) at five time points (3, 6, 12, 24, and 72 h). We detected DEGs under abiotic stresses compared with the untreated control: cold (10,718 DEGs), heat (9,990 DEGs), mannitol (3,548 DEGs), and salt (5,766 DEGs). We identified 29, 26, 12, and 20 bZIP DEGs in pepper in response to cold, heat, mannitol, and salt treatment, respectively (Supplementary Figure 3; Supplementary Table 4). The bZIP DEGs primarily belonged to specific subgroups as follows: A (11), D (10), E (8), G (6), and S (16). This indicates that *bZIP* genes in these subgroups may have roles in responding to abiotic stresses. Several functional *bZIP* genes in pepper, such as *CabZIP25* (CANN_61) and *CaBZ1* (CANN_67), were significantly upregulated under abiotic stress, which was consistent with previous studies (Moon et al., 2015; Gai et al., 2020) and validated the accuracy of our transcriptome analyses. We performed a temporal soft-clustering analysis of whole DEGs in pepper to investigate the expression patterns of *bZIP* and other genes in pepper (Figure 4A). DEGs were grouped into four distinct clusters (C1–C4) based on expression levels under each abiotic stress. We observed that the bZIP DEGs were abundant in C3 and C4 under cold stress, C1 and C3 under heat stress, C1 and C2 under mannitol stress, and C1 and C4 under salt stress (Figure 4B). This suggests that *bZIP* genes in these clusters were associated with specific functions under these abiotic stress conditions.

We conducted GO enrichment analysis to predict the potential functions of *bZIP* genes in pepper using the genes in the selected eight clusters including abundant bZIP DEGs (Figure 4C). When we surveyed which GO terms were enriched in the eight clusters, a variety of functional descriptions were identified and many were distinctly observed in specific clusters (Figure 4C). For example, heat stress C1 contained GO terms in cellular component categories such as “cellular anatomical entity,” “organelle,” and “intracellular organelle.” By contrast, heat stress C3 contained GO terms in biological process categories such as “response to oxidative stress,” “response to temperature stimulus,” and “response to abiotic stimulus.” This result suggests that *bZIP* genes in pepper were associated with diverse functions under abiotic stresses, and their functions differed among the clusters. We investigated response-related GO terms in each cluster to verify specific functions in response to abiotic stress, as the previous study focused on response-related GO terms under abiotic stress conditions (Liu et al., 2015; Supplementary Figure 4). The repertoires of abiotic stress response-related GO terms were distinct from those of expression clusters. For example, salt stress C1 with abundant *bZIP* genes in subgroup A (bZIP_only-enriched subgroup) and salt stress C4 with abundant *bZIP* genes in subgroup D (DOG1-enriched subgroup) had different GO term repertoires such as “response to abscisic acid” and “response to stress,” respectively. *CabZIP25* of pepper in subgroup A was highly expressed under salt stress and associated with ABA signaling (GO:0009737), thereby enhancing resistance to stress (Gai et al., 2020). The *OsHBP1b* of *O. sativa* in subgroup D promoted salt stress tolerance by altering activation of the ROS scavenging system, and had the child GO term of “response to stress” (GO:0006952; Lakra et al., 2015). The biotic stress-related GO terms (e.g., “response to biotic stimulus,” “response to external biotic stimulus,” and “response to other organisms”) were only enriched in salt stress C4, which was a typical function of *bZIP* genes in subgroup D (Alves et al., 2013; Fu and Dong, 2013; Droge-Laser et al., 2018). These results represent that bZIP_only and bZIP_IDs were functionally diverse according to the diverse *bZIP* gene architectures, which resulted from the integration of additional domains. Our analyses provided novel insights into *bZIP* gene expression and function in pepper, which will facilitate further studies.

### Expression and functional prediction of tomato *bZIP* genes under abiotic stress

**Figure 5:**
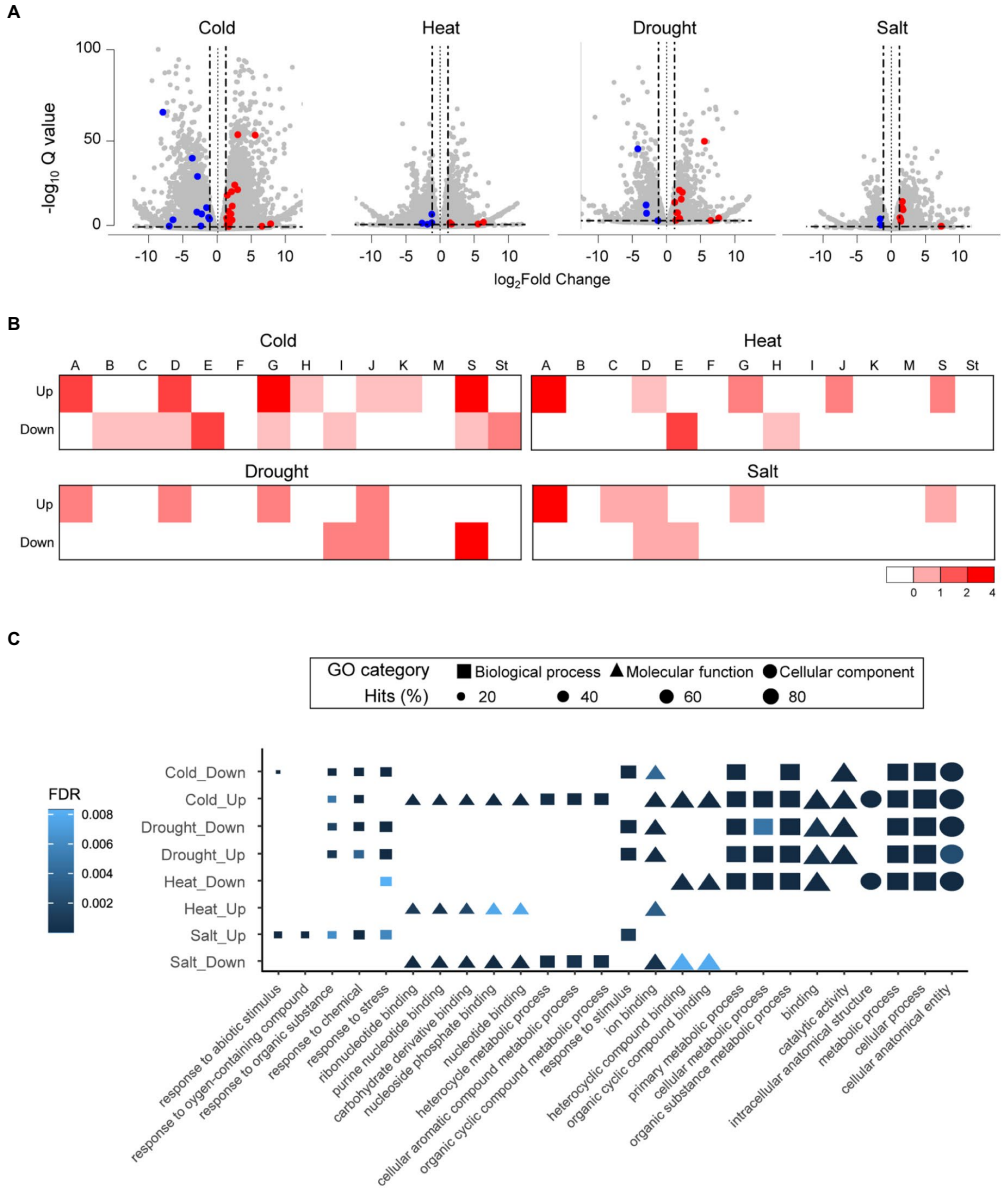
Expression analyses and functional predictions of differentially expressed genes under different abiotic stresses in tomato (*Solanum lycopersicum*). **(A)** Volcano plot for tomato DEGs under different abiotic stresses. Scattered gray dots represent whole genes in tomato. Red and blue dots depict upregulated and downregulated bZIP DEGs based on 1-fold expression difference (represented by two black vertical lines). **(B)** Number of bZIP DEGs in each subgroup is displayed in a heatmap. Heatmap values on the bottom right side indicate the number of bZIP DEGs in each subgroup. **(C)** Dot plots depict the top five GO enrichment results for each group including *bZIP* genes. Shapes represent the GO categories; shape sizes represent the GO term frequencies.

We analyzed the expression profiles of whole genes in tomato (*S. lycopersicum*) along with the newly annotated *bZIP* genes to predict the function of tomato *bZIP* genes under abiotic stress. The gene expression profiles were examined using RNA-seq data under four stresses: cold, drought, heat, and salt. A total of 9,251 (cold), 1,174 (drought), 4,632 (heat), and 1,520 (salt) DEGs were identified. We identified 28, 15, 8, and 9 bZIP DEGs in tomato under cold, drought, heat, and salt conditions, respectively (Figure 5A; Supplementary Table 5). Subgroups A (11), D (8), E (7), G (9), and S (10) were observed as dominant subgroups with bZIP DEGs at least under one or more stress conditions, which were the same as those in pepper, suggesting that Solanaceae family members share common subgroups associated with abiotic stress responses. We verified that *SlbZIP38* (SLYC_44) expression was downregulated under salt stress, which was consistent with the previous study and validated the accuracy of our expression analyses of tomato *bZIP* genes (Supplementary Figure 5; Pan et al., 2017). We examined the expression levels of bZIP and other genes in tomato under abiotic stress and classified DEGs as upregulated or downregulated (Figure 5B). The results showed that all bZIP DEGs in subgroup A were upregulated, consistent with previous studies reporting that they were highly expressed under abiotic stress to regulate ABA-dependent signaling (Yoshida et al., 2010; Gai et al., 2020).

Next, we conducted a GO enrichment analysis of *bZIP* gene functions in the eight groups containing bZIP DEGs in tomato (Figure 5C). We observed that several functional descriptions appeared in specific groups, similarly observed in pepper. For example, metabolism-related GO terms such as “metabolic process,” “organic substance metabolic process,” and “primary metabolic process” existed in five groups (cold-downregulated, cold-upregulated, drought-downregulated, drought-upregulated, and heat-downregulated), and binding-related GO terms were enriched in three groups (cold-upregulated, heat-upregulated, and salt-downregulated). These results suggest that *bZIP* genes were involved in diverse functions under abiotic stresses, and these functions were distinct among the groups. We also examined GO terms related to response and metabolism (GO:0050896 and GO:0008152, respectively) in each group (Supplementary Figure 6). The repertoires of enriched GO terms for response and metabolism were distinct among the groups. GO term repertoires specifically differed between two groups: cold-upregulated with abundant *bZIP* genes in subgroup G (MFMR-enriched subgroup), and salt-upregulated with many *bZIP* genes in subgroup A (bZIP_only-enriched subgroup). The cold-upregulated group included abundant metabolism-related GO descriptions, whereas the salt-upregulated group primarily contained response-related GO terms. We did not find functional *bZIP* genes related to those GO terms in subgroups G and A in Solanaceae genomes including tomato but did verify those functions of *bZIP* genes in other plant genomes. For example, *GBF1* of *Z*. *mays* in subgroup G functioned in metabolism (GO:0006355) for cold stress tolerance by binding to a pseudo-palindromic *cis*-acting element called G-box region of alcohol dehydrogenase-1 (Devetten and Ferl, 1995; Shi et al., 2017). The *A. thaliana AtbZIP37* in subgroup A was involved in ABA-dependent signaling under high salinity conditions (GO:0009651), thereby enhancing stress resistance (Yoshida et al., 2010). These results suggest that bZIP_only and bZIP_IDs had diverse functions under different abiotic stress conditions in tomato due to distinct domain architectures.

## Conclusion

The *bZIP* genes are an essential transcription factor that regulates plant growth. We updated the annotation of *bZIP* genes and performed comprehensive comparative and functional analyses of the updated *bZIP* genes in nine Solanaceae genomes and *A. thaliana*. We divided *bZIP* genes into bZIP_only and bZIP_IDs depending on the domain architectures, and comparatively analyzed the two groups. Our data identified distinct motif compositions in bZIP_only and bZIP_IDs primarily due to the upstream (bZIP_only) or downstream (bZIP_IDs) locations of specific motifs relative to the bZIP domain. Based on the phylogenetic relationship, we found that bZIP_only and bZIP_IDs were clustered into distinct subgroups with unequal copy numbers among the subgroups. These results suggest that bZIP_only and bZIP_IDs underwent unequal copy number expansion in specific subgroups since their initial emergence from ancestral genes. Transcriptome analyses with GO enrichment analysis in pepper (*C. annuum*) and tomato (*S. lycopersicum*) revealed the potential functions of *bZIP* genes interacting with other genes under various abiotic stress conditions. Our data suggested functional diversity and distinct roles for bZIP_only and bZIP_IDs under four abiotic stress conditions. These results may be due to the distinct gene structures resulting from domain integration, which ultimately contributed to the functional diversification of *bZIP* genes.

Previous studies suggested that gene family analyses using advanced annotation methods could provide novel insights into the structural and functional characteristics of genes (Bayer et al., 2018; Chae et al., 2021; Lee et al., 2021; Guk et al., 2022). We also performed independent annotation updates and comparative analyses of *bZIP* genes in Solanaceae. In contrast to previous studies of *bZIP* genes, we focused on an extensive comparison of *bZIP* genes in Solanaceae to understand *bZIP* gene characteristics in the Solanaceae family rather than in individual species. Specifically, our data revealed comprehensive structural and expressional differences between two types of domain architectures: bZIP_only and bZIP_IDs, and suggested that those two architectures are involved in diverse functions under abiotic stress conditions. Taken together, our analyses provide novel insights into the structural, evolutionary, and functional features of *bZIP* genes in the Solanaceae family. These results will facilitate future research in plant *bZIP* genes.

## Data availability statement

The datasets presented in this study can be found in online repositories. The names of the repository/repositories and accession number (s) can be found in the article/Supplementary material.

## Author contributions

SK designed the study. J-WC, H-EK, and SK performed the re-annotation and comparative analyses and edited and reviewed the final version. J-WC wrote the initial manuscript draft. All authors contributed to the article and approved the submitted version.

## Funding

This study was supported by a National Research Foundation of Korea (NRF) grant funded by the Korean government (NRF-2022R1C1C1004918) to SK and the Korea Institute of Planning and Evaluation for Technology in Food, Agriculture, and Forestry (IPET) through the Digital Breeding Transformation Technology Development Program funded by Ministry of Agriculture, Food, and Rural Affairs (MAFRA; 322075–3) to SK.

## Conflict of interest

The authors declare that the research was conducted in the absence of any commercial or financial relationships that could be construed as a potential conflict of interest.

## Publisher’s note

All claims expressed in this article are solely those of the authors and do not necessarily represent those of their affiliated organizations, or those of the publisher, the editors and the reviewers. Any product that may be evaluated in this article, or claim that may be made by its manufacturer, is not guaranteed or endorsed by the publisher.

## Footnotes

^1^ http://pfam.xfam.org

^2^ https://weblogo.berkeley.edu/logo.cgi

^3^ https://www.biobam.com/omicsbox

^4^ http://itol.embl.de

^5^ https://www.biobam.com/omicsbox

## Abbreviations

IDs: Integrated domains
GO: Gene ontology
FPKM: Fragment per kilobase of transcript per million mapped reads
DEGs: Differentially expressed genes
FDR: False discovery rate
