# Supplementary material for: Two different domain architectures generate structural and functional diversity among bZIP genes in the Solanaceae family
Source: Front Plant Sci. 2022 Aug 19;13:967546. doi: 10.3389/fpls.2022.967546 (PMC9437623; doi:10.3389/fpls.2022.967546)
Supplement: Supplementary file 1 [file Data_Sheet_1.PDF]

Supplementary Figure 1

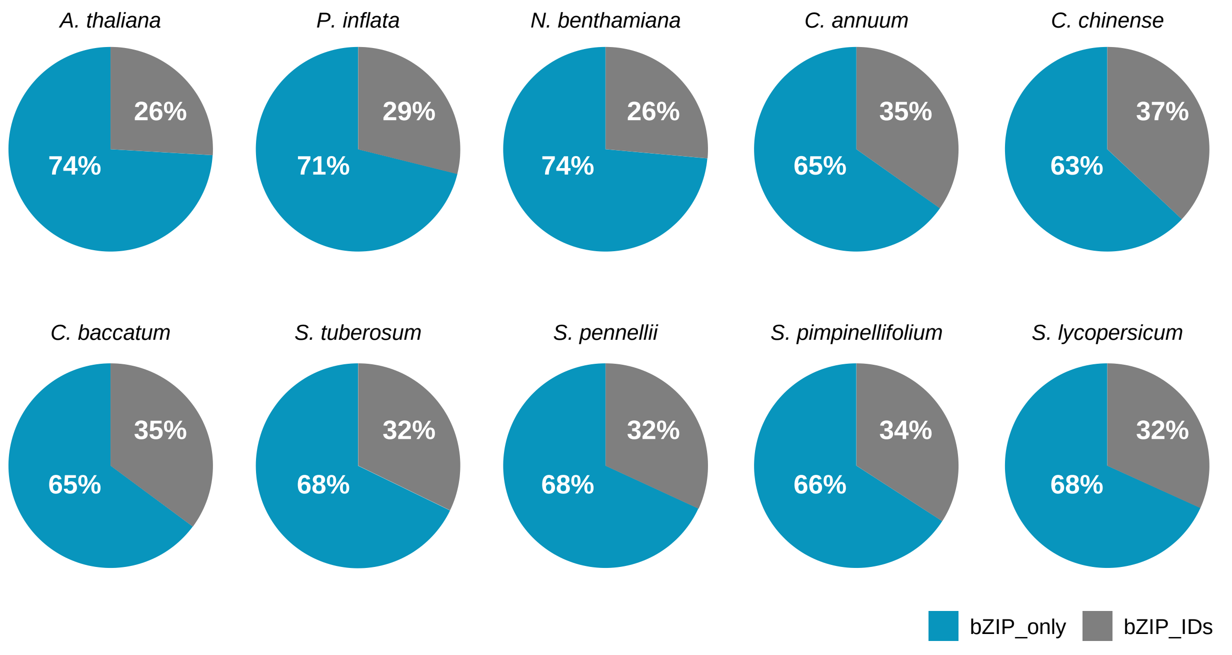

FIGURE S1 | Percentages of bZIP_only and bZIP_IDs in each species. The colors of the pie chart indicate bZIP_only (blue) and bZIP_IDs (gray). The species name is listed above each pie chart.

Supplementary Figure 2

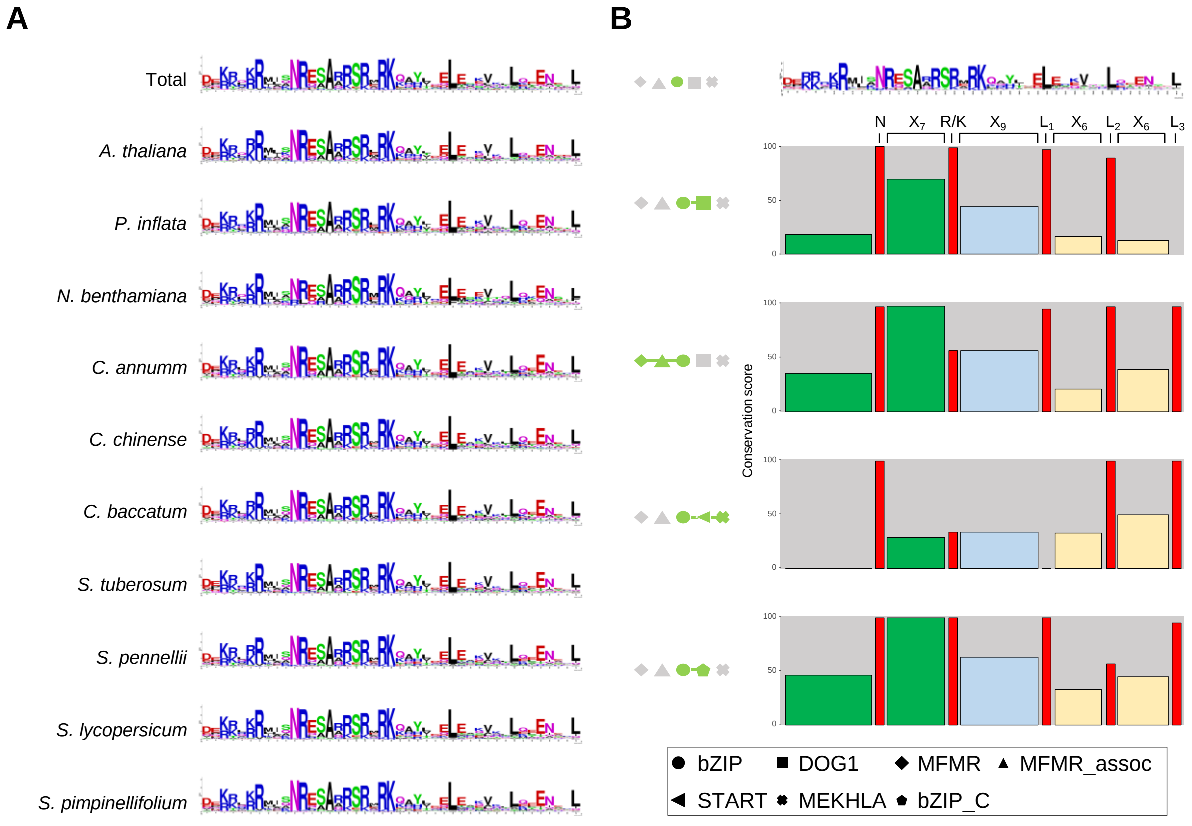

FIGURE S2 | Amino acid sequence composition of the bZIP domain. **(A)** Amino acid sequence composition of the bZIP domain in each species. The relative frequencies of individual amino acids in the bZIP domain from 10 plant genomes are illustrated by the height of the residue. **(B)** Conservation score of the top four domain architectures compared to amino acid sequences in the bZIP domain of bZIP_only. The top five domain architecture repertories are shown on the left of the chart as different geometrical symbols. The conservation scores of each compartment and significant residues are represented as bar plots. Green, light blue, and yellow bars represent three bZIP domain regions: basic region, hinge region, and leucine zipper region, respectively. The amino acid residue abbreviations in black above the bar plots indicate significantly conserved residues in bZIP genes.

Supplementary Figure 3

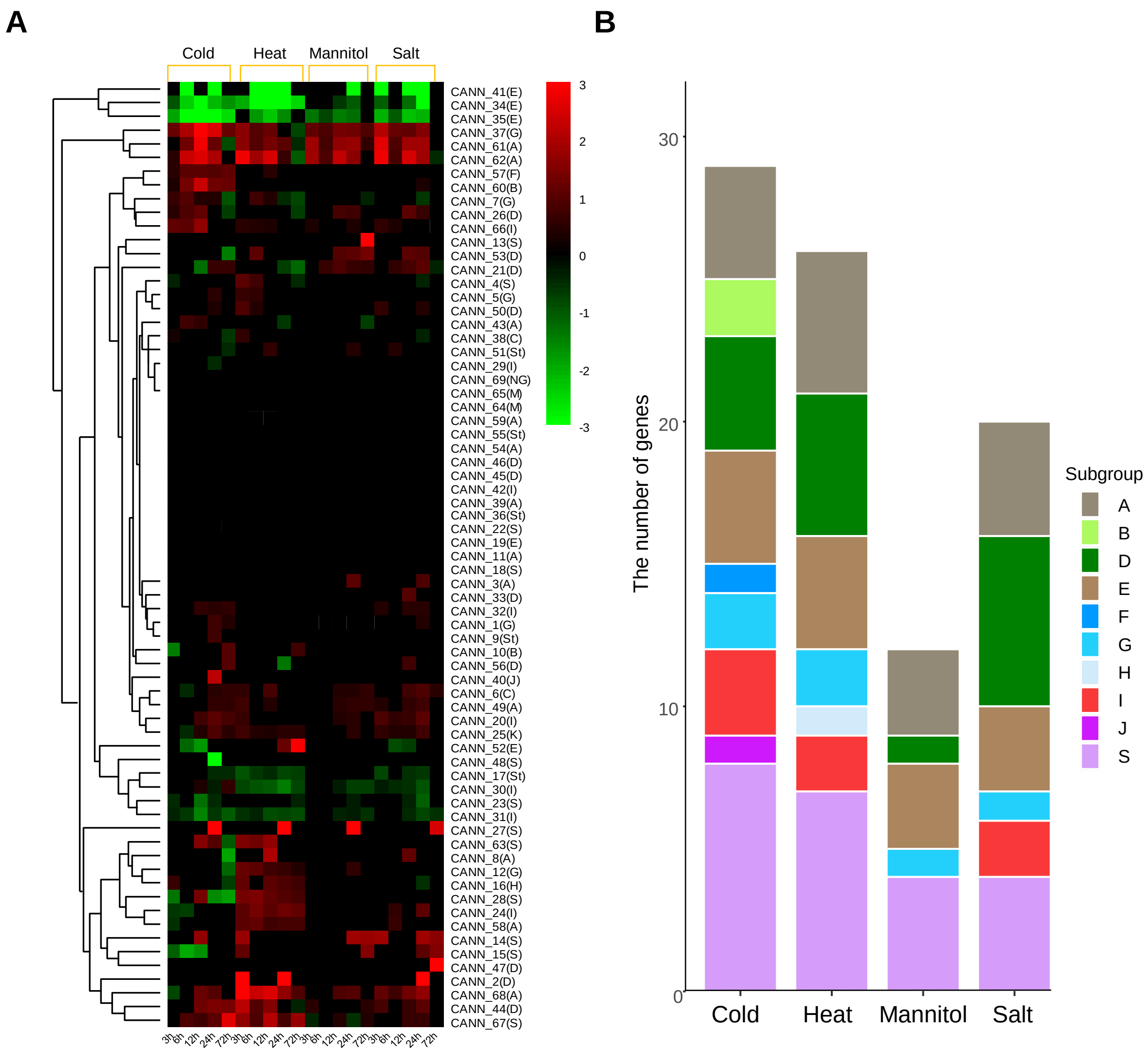

FIGURE S3 | Differential expression of bZIP genes in pepper under various abiotic stresses. **(A)** Log_2 (fold-change) values were calculated as FPKM (abiotic stress) / FPKM (control) and plotted as a heat map. Expression values are adjusted to a maximum of 3 and a minimum of 3 for effective visualization. The gene name is listed next to the heatmap with the subgroup information in parentheses. Different time points of each stress are indicated at the bottom of the heat map. The color scale bar located at the upper right side of the heat map represents normalized expression values: red indicates high expression level, and green indicates low expression level. **(B)** Numbers of bZIP DEGs in the subgroups under four abiotic stresses. Bar colors indicate subgroups.

Supplementary Figure 4

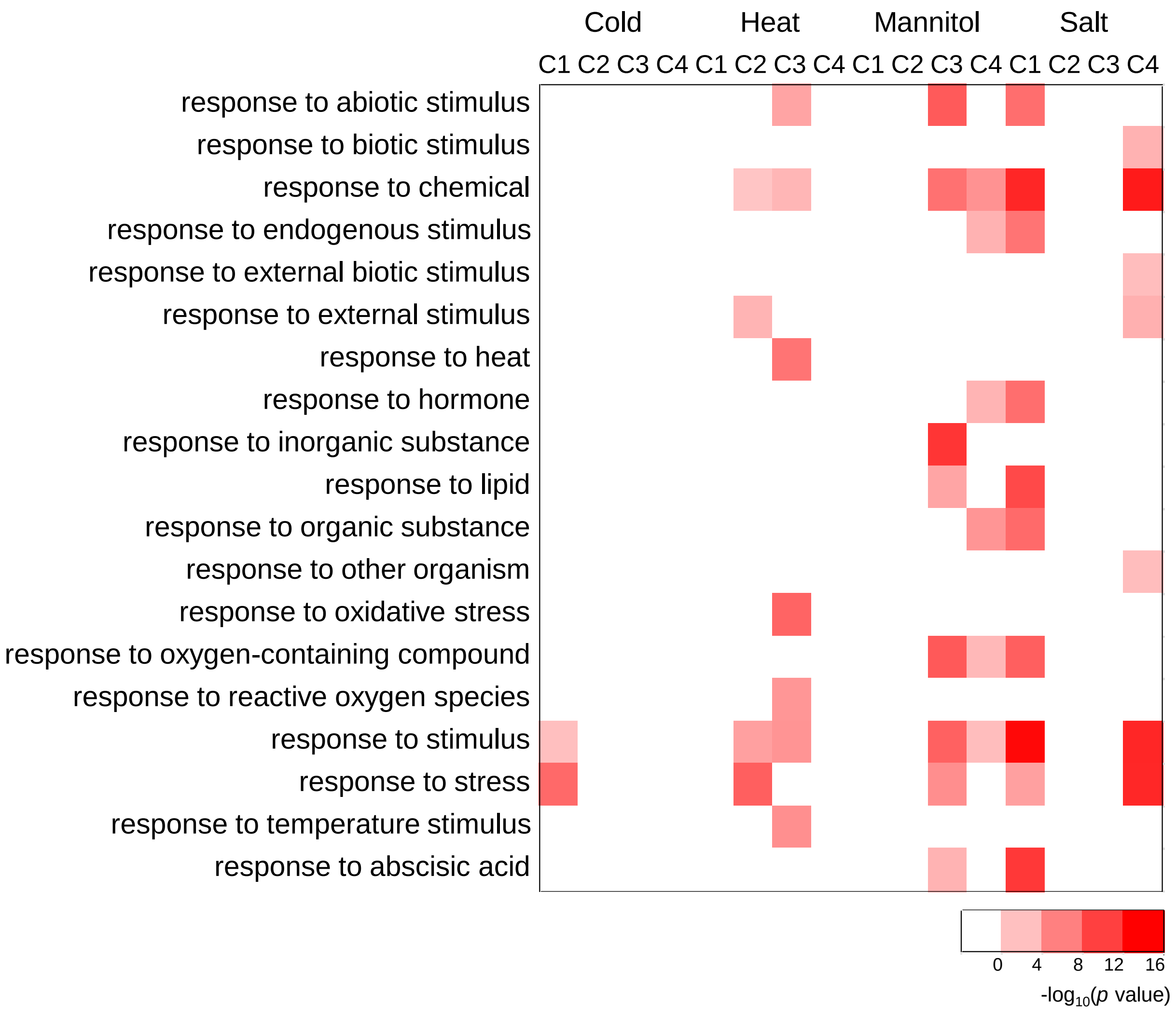

FIGURE S4 | Enriched response-related gene ontology terms under four abiotic stresses. Each stress and cluster are listed above the heat map. The list of GO descriptions is given at the left of the heatmap. Heatmap values represent the relative *p*-value significance by Fisher's exact test (color scale bar below the heat map).

Supplementary Figure 5

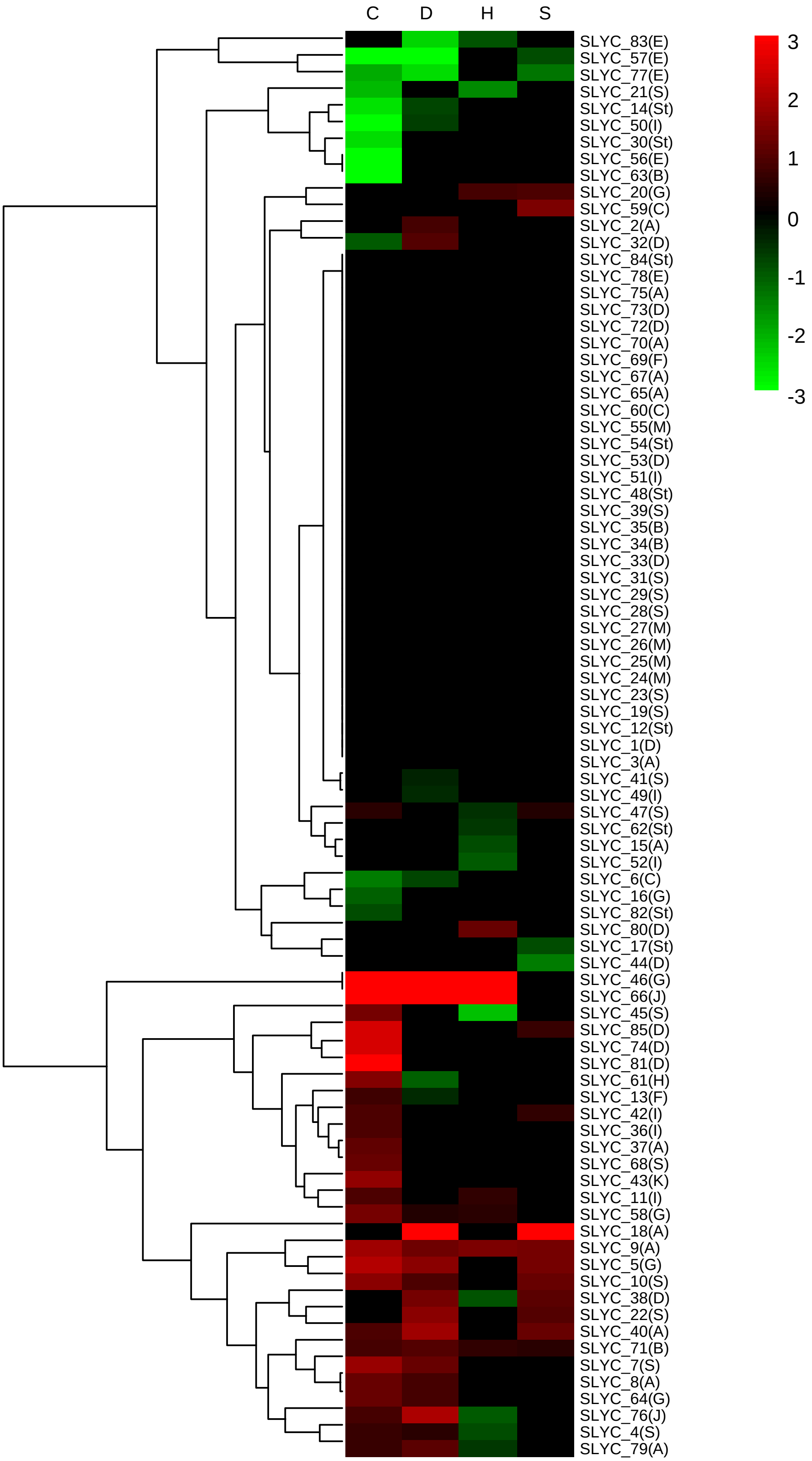

FIGURE S5 | Expression profiling of tomato bZIP genes using RNA-seq data under various abiotic stresses. Log_2 (fold-change) of tomato bZIP genes comparing FPKM in each abiotic stress treatment to FPKM in the control is represented as a heat map. For optimal visualization, expression values are set to a maximum of 3 and a minimum of 3. Color scale at the upper right side of the heatmap shows a range of normalized expression levels from –3 (green) to 3 (red). Gene names and subgroups are listed on the right of the heat map.

Supplementary Figure 6

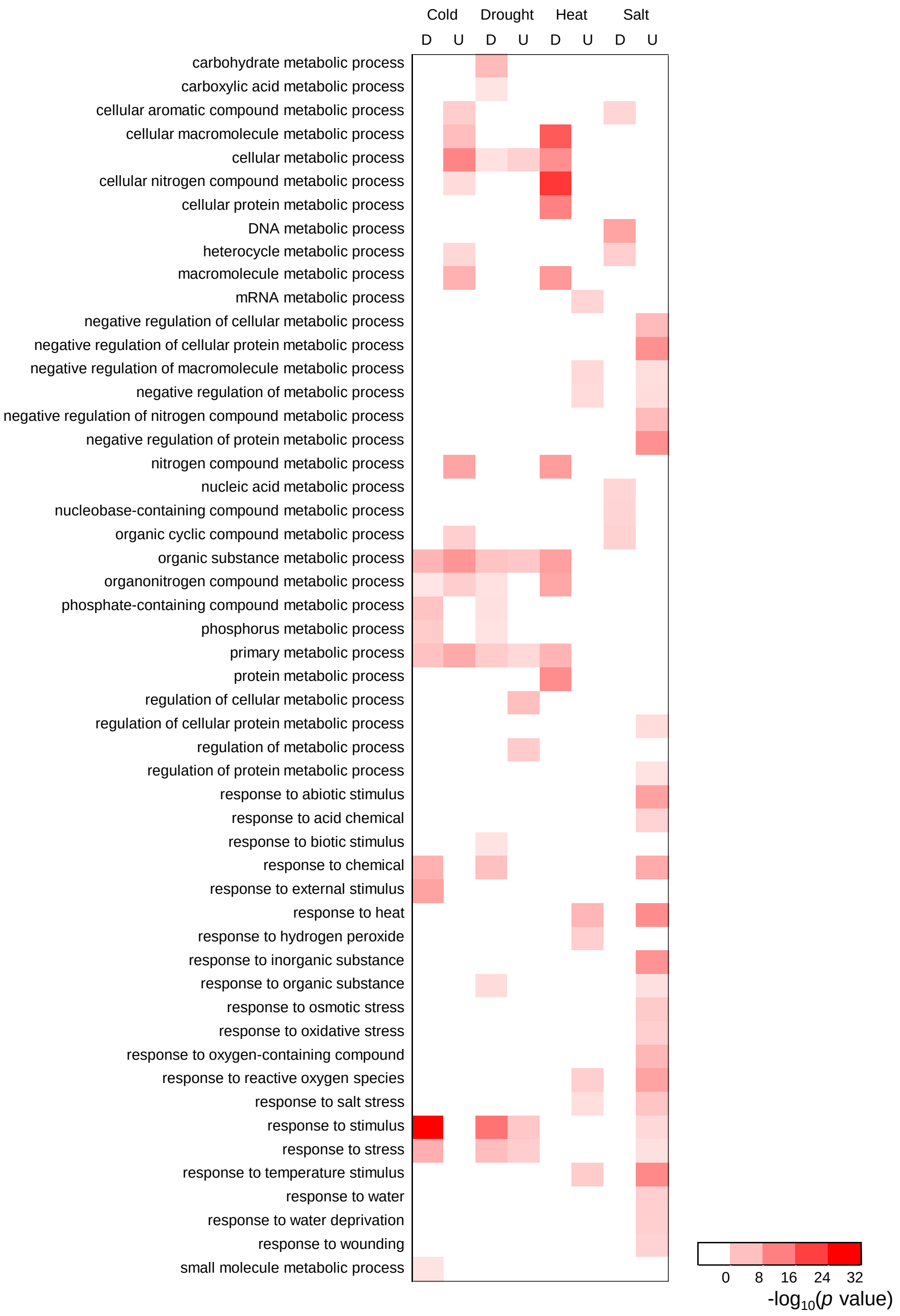

FIGURE S6 | Heat map showing *p*-value significance of enriched metabolism-related and response-related GO terms in each group of tomato. D and U above the heat map indicate downregulated and upregulated groups. The list of GO terms is located at the left of the heatmap. Fisher's exact test was used to determine the *p*-value significance, which is indicated in the color scale bar at bottom right.
